# Environmental Sustainability Commitment and Financial Performance of Firms Listed on the Johannesburg Stock Exchange (JSE)

**DOI:** 10.3390/ijerph17207504

**Published:** 2020-10-15

**Authors:** Obey Dzomonda, Olawale Fatoki

**Affiliations:** Department of Business Management, Turfloop Campus, University of Limpopo, Private Bag X1106, Sovenga 0727, South Africa; olawale.fatoki@ul.ac.za

**Keywords:** environmental sustainability commitment, financial performance, firms, sustainable development, Johannesburg Stock Exchange, South Africa

## Abstract

The importance of heeding the environmental sustainability commitment call cannot be underestimated. Laggards in terms of environmental sustainability commitment are likely to face fines and penalties as talks to tighten environmental legislation are now at an advanced stage globally. The current work assessed the link between environmental sustainability commitment and financial performance of firms listed on the Johannesburg Stock Exchange (JSE). The study was quantitative in nature with a case study research design. The longitudinal design was adopted where the researcher collected panel data from 2011–2018. The population of the study included all firms listed on the JSE Responsible Investment Index in South Africa. The sample constituted of 32 firms listed on the Financial Times Stock Exchange FTSE/JSE Responsible Investment Index in South Africa. The researchers employed the panel regression analysis model to analyze the data. Specifically, the Feasible Generalized Least Squares regression model was used in this study. Financial performance was treated as the dependent variable as measured by earnings per share and share price. The independent variables of the study included components of environmental sustainability such as carbon emission reduction and environmental compliance. Control variables such as firm size and liquidity were used in the study. The findings indicated that carbon emission reduction was positively and significantly related to earnings per share and share price. The findings further exhibited that environmental compliance was positively related to earnings per share and share price. It was concluded that firms can enhance their financial performance from environmental investment as all the hypotheses were supported. This study contributes practically towards shaping environmental policies and it also serves as motivation to listed companies that they can enhance both their profitability and market value from environmental investments.

## 1. Introduction

The issue of environmental sustainability commitment has become a famous topic recently [1] and it is highly emphasized in the sustainable development goals (SDGs) on both agenda 2030 and 2063 [2]. Sustainability can be summarized into three pillars which are economic, society and the environment [3]. The focus of the current study is on environmental sustainability practices of firms listed on the Johannesburg Stock Exchange (JSE). The environment plays a key role as it supplies all the resources required to sustain all human activities [4]. There is a serious need to adopt a proactive stance towards protecting the environment [5]. This is because of the reported cases of climate change which have long been felt globally. In South Africa, other catastrophes such as floods and heat waves have been felt due to climate change emanating from irresponsible environmental behavior. There are also fears that if carbon emissions are not monitored, it may result in serious increase in temperatures which may stand as a threat to humanity. As such, pressure is burgeoning every day for businesses to consider environmental sustainability seriously [6]. The logic behind this is that listed firms contribute extensively towards environmental pollution from their business activities such as mining, transport, and heating of offices.

Nevertheless, listed firms are responding slowly to the call for environmental sustainability commitment in terms of compliance and reporting [7]. Consequently, some firms are involved in fraudulent activities to escape environmental commitment [8], while others are involved in greenwashing [9]. On that backdrop, it is noted that the number of highly sustainable firms remain low as compared to firms that do not commit to environmental sustainability initiatives [10]. The unsatisfactory environmental sustainability commitment among firms is worrying given that firms contribute towards environmental damage [2]. A report by International Energy Agency (IEA) [11] indicates that between 2008 and 2013, manufacturing, construction, and energy firms emitted approximately 66.72% of total global carbon emissions. Furthermore, South Africa tops the list of greenhouse gas emitters as approximately 95% of its electricity comes from coal [12]. South Africa is indeed on the brink of a sustainability threat if environmental variables such as carbon emission and environmental compliance are not efficiently managed [13].

The issue of sustainability commitment and performance are key aspects for listed firms to increase the value of their shares, and hence attract investors [14]. This is so because of the emergence of responsible investing where institutional investors assess the sustainability of a business before committing their investments [14]. Firms actively committed to environmental sustainability can achieve improved growth and cost savings, hence a health financial standing [11]. Nevertheless, the relationship between environmental sustainability commitment and financial performance remains indistinct [15]. Some studies point out that environmental sustainability commitment positively influences the financial performance of a firm through operational efficiency, which consequently cut down costs [16,17,18]. Conversely, another strand of literature posits that environmental sustainability initiatives deplete funds that could have been used to fund other profitable investments, hence, negatively affecting financial performance [19,20]. Surprisingly, another strand of scholars indicate that environmental sustainability commitment has no effect on financial performance [21]. 

Based on the above ongoing debate, there is need for new empirical studies to resolve the inconclusiveness of findings [22]. Studies that have investigated the effect of environmental sustainability commitment on financial performance are limited and underdeveloped in developing countries [18]. Against this backdrop, the purpose of this study is to empirically evaluate the effect of environmental sustainability commitment (carbon emission reduction and environmental compliance) on financial performance of JSE listed firms. The study contributes to existing literature in several ways. For example, the study improves the environmental sustainability construct by adding a new variable such as environmental compliance which have been neglected in existing literature yet very crucial if environmental sustainability is to be attained. The argument is that attaining environmental compliance can help firms to reduce emissions, hence, reducing ozone layer depletion. Additionally, the study also adopts a multi-dimensional model of financial performance, which incorporates both the accounting measures of financial performance (earnings per share) and market-based measures (share price). This has been missing in existing literature in South Africa.

### 1.1. Literature Review

#### Stakeholder Theory

The stakeholder theory as propounded by Freeman [23] is a strategic management concept which argues that corporate managers should strive to respond and consider the interest of key stakeholders in their business network. On that note, it is argued that a business does not operate in a vacuum but rather in a network with different stakeholders all concerned on how the corporate conducts its business activities. Freeman [23] argues that a firm should go beyond profit maximization and consider building relations with its key stakeholders. His view is against the traditional shareholder theory that only prioritizes shareholders of the entity leaving some other parties’ interests unattained. Rokhmawati, Gunardi and Rossi [24] warn that firms that do not incorporate stakeholders’ interests in their strategies risk chances of business failure in the long run. According to Freeman [23], a stakeholder is defined as an individual or group of people who can affect or be affected by the business’ operations. Dodson, Azevedo, Mohiuddin, Defavari and Abrahão [25] assert that organizations should do stakeholder analysis to identify key stakeholders and their interests in the organization. Stakeholder analysis assists a firm to clearly identify and manage different needs of all key stakeholders to avoid future conflicts and lawsuits. Dodson et al. [25] argue that stakeholder analysis is important in order to accommodate special cases such as the natural environment, which is in most cases, neglected because it does not reflect in company financial statements. Stakeholders can be grouped into either primary or secondary stakeholders [25]. Primary stakeholders are defined as parties that have a direct link and influence the day to day running of the business. These include; customers, suppliers, shareholders and investors [26]. Bad relations or conflicts with this inner circle affects business transactions instantly. Secondary stakeholders include parties that are indirectly linked to the business, but their actions influence the business. Among others, these include the government, the media, and other regulatory bodies. Even though the stakeholder theory has received some criticism [27,28], it remains widely adopted it sets precedence to understand environmental sustainability commitment among firms by responding to stakeholder interests through environmental investments. 

### 1.2. Environmental Sustainability

The Organization for Economic Co-operation and Development (OECD) [29] defines environmental sustainability based on the following criteria: regeneration, substitutability, assimilation, and irreversibility. In this study, environmental sustainability is defined as proactive strategies designed to protect the environment by reducing carbon emissions and being environmentally compliant. The issue of environmental sustainability has dominated the agendas of environmental concerned bodies globally [30]. Discussions about the environmental sustainability intensified post 2015 [31] due to the concern over environmental degradation [32]. Most developed countries have already started investing seriously on sustainability issues [10]. However, African states have been identified as laggards when it comes to the adoption of the environmental sustainability agenda [33]. There is still heavy reliance on fossil fuels in Africa regardless of rising concerns over environmental damage [31]. Fears are that if firms do not commit to environmental sustainability activities such as carbon emission reduction and environmental compliance, the level of ozone depletion will be unmanageable. Large firms have been blamed for causing excess environmental damage [20]. More often than not, firms are involved in fraudulent activities to escape environmental commitment [34]. Regardless of environmental policies that are in place for firms to comply with, a significant number of them still evade compliance [35]. A good firm strategy is no longer characterized by its ability to boost profits only, but also on how it caters for the environment [10]. Environmental sustainability is a strategic issue which requires a firm to produce a profound environmental sustainability commitment strategy [10]. The environmental sustainability commitment strategy implies that the firm undertakes to generate value from its business activities by being environmentally sensitive and being actively involved in the issues thereof [36]. An environmental sustainability strategy should be integrated into the main business strategy rather than being treated as a separate strategy [37]. In this study, this entails incorporating carbon emission reduction and environmental compliance into the main business strategy. 

#### 1.2.1. Carbon Emission Reduction 

Greenhouse gas emissions remain one of the top challenges faced globally since greenhouse gases are linked to climate change and ozone layer depletion [38]. On that note, the issue of carbon emissions requires agent attention globally [39] because reducing carbon emissions may determine the attainment of the rest of other SDGs [38]. It is imperative to devise a consortium of strategies to cut down carbon emissions. However, regardless of vowing to cut down emissions to 42% by 2025 following the Paris Agreement on Climate Change in 2016 [40], South Africa remains one of the worst emitters in the world, where the average CO_2_ emission per person doubles the world CO_2_ emission average [41]. There are fears that if the issue of climate change is not mitigated, South Africa is likely to be twice warmer than the global average [40]. The problem of high CO_2_ emissions in South Africa is linked to overreliance on coal as the major resource to generate electrical energy [42]. The issue of climate change is a reality than a myth. Hence, there are trepidations that if temperatures can rise again by a 2 °C, the issue of climate change will be uncontrollable [43].

#### 1.2.2. Environmental Compliance

The concern over the depletion of natural resources coupled with the goal to reduce emissions have seen more environmental policies, standards and regulations being implemented globally [44]. In some industries such as construction and retail, firms are under pressure from customers to possess environmental standard certificates [45]. Environmental non-compliance poses a serious threat to the firm. These manifest in the form of lawsuits, penalties, and suspension of trading licenses. For instance, lack of compliance negatively affected Enterprise Foods in South Africa when the company’s food items were linked to the outbreak of Listeriosis. This led to a serious outcry and protests by civilians, leading to serious consequences for the firm. Recently, institutions globally have been calling for stringent penalties on firms that fail to account for their environmental impact. Due to the rapid adoption of new technologies and leveraging on innovation, the conventional thinking that environmental compliance increases cost has since lost support [1]. Environmental compliance plays a crucial role in the survival of a firm [44]. However, issues related to lack of accountability, transparency and participation are among key factors hindering progress in the environmental sustainability agenda [38]. Serious environmental compliance is required to attain environmental sustainability. Environmental compliance at firm level is exhibited through the presence of internal environmental policies, possession of environmental excellence certificates, adoption of environmental certifications such as the International Standards Organization 4001 and the absence of environmental fines and penalties [46].

### 1.3. Relationship Between Environmental Sustainability Commitment andFfinancial Performance

#### 1.3.1. Relationship Between Carbon Emission Reduction and Financial Performance

Existing literature about carbon emission reduction and firm financial performance nexus is inconclusive [24]. Cucchiella, Gastaldi and Miliacca [39] assessed carbon emission reduction and the effect on profitability. The study used large Italian firms. The results indicated that cutting down carbon emissions offers a business sustainable benefits. Yu and Tsai (2018) [47] concur and allude that efforts by a firm to cut down its carbon emissions improves its longevity and sustainability. This is because it is now crucial than ever to excel in environmental performance. Lewandowski [48] assessed whether emission reductions influenced the profitability of firms using 1640 international firms between 2003 and 2015. Lewandowski [48] reported that emission reductions were directly linked to return on sales. The effect was stronger for companies which excelled in carbon emission reduction initiatives compared to the laggards. Capece, Di Pillo, Gastaldi, Levialdi, Miliacca [49] scrutinized the link between reduction in emissions and financial performance of Italian firms. The results indicated that firms actively involved in initiatives to cut down emissions showed positive financial returns. 

However, another line of thought adopts the traditional economics view that environmental participation is costly to the firm. Hence, the argument is that investing in technology and systems to cut carbon emissions can reduce the profits of the firm [19]. In support of this view, Rokhmawati et al. [24] reported that high emissions were directly related to profitability. This entails that firms still make gains from using fossil fuels because the legislation is too weak to make firms cut down their emissions. Hence, in the absence of a strong environmental legislation other firms may continue to make profits from unsustainable sources to power their industries and business operations at the expense of the environment. Rokhmawati and Gunardi [50] analyzed how emissions are related to financial performance of listed Indonesian firms. The study established that excessive emissions were directly linked to superior FP. The study made an important analysis that in the case where implementing carbon emission reduction is expensive than non-participation, firms end up not heeding the call to cut down emissions. Hence, the argument was that environmental regulation should be executed and laggards should be punished if all firms are to participate in environmental protection initiatives. 

The last strand of literature dismisses the assertions that carbon emission reduction influences financial performance. For instance, Worae and Ngwakwe [51] assessed the effect of environmental investments on a firms’ financial prospects using listed companies and data from 2008–2014. Employing the Granger causality analysis, the results showed a unidirectional causal relationship. The authors argued that sometimes environmental sustainability commitment may not influence the profitability of a firm in any manner if it is not one of the key success factors in that particular industry. In that case either a weak or strong environmental commitment may not produce any result either positive or negative. Misani and Pogutz [52] also established that emission reduction had no effect on the profitability of a firm. Having used carbon intensive firms from 2007 to 2013 and testing emission reductions against the Tobin’s Q, the findings suggested that investors will be indifferent on whether to invest in future or not. The study also noted that a firm is likely to record enhanced financial performance when carbon emission reduction is moderate as compared to when it is low or high. 

Regardless of the inconclusiveness of existing findings, this study proposes that investing in technologies which cut carbon emissions increases the financial performance of firms. This is because firms can eliminate inefficiencies in their production systems which can result in positive earnings per share (EPS) and possibly an improvement in the share price emanating from investors attracted by firms which commit seriously towards environmental protection. Moreover, the authors of this study are of the view that listed firms can earn green trust from green oriented stakeholders which enhances the value of their shares. Alternatively, managing to reduce carbon emission may mean the firm could have adopted other renewable sources of energy such as solar, hydroelectricity, and biogas which tend to be cheaper in the long run. This means the firm can manage to save on costs which enhances EPS. Based on the above evidence, the following hypotheses are proposed:

**Hypothesis** **Ha_1_.** *There is a significant positive relationship between carbon emission reduction and the EPS of firms listed on the JSE.*

**Hypothesis** **Ha_2_.** *There is a significant positive relationship between carbon emission reduction and the share price of firms listed on the JSE.*

#### 1.3.2. Relationship Between Environmental Compliance and Financial Performance

The effect of environmental compliance on financial performance is indistinct [53]. There is a need for more empirical studies to demystify this relationship especially from an emerging market perspective since the institutional environment might differ from that of developed countries so is the effect on financial performance [1]. Another strand of literature submits that a firm is likely to avoid costs which come with fines and penalties emanating from non-compliance. Even though the cost of maintaining an environmentally compliant status might be relatively high, fines and penalties emanating from environmentally irresponsible behavior can be unbearable [54]. For instance, BP is still haunted by a case emanating from the 2010 oil spill in the Gulf [55]. Such a penalty can leave a firm with serious cash flow problems. Furthermore, a firm is likely to avoid penalties and fines if they comply with environmental policies and standards such as the International Organization for Standardization 4001 standards [56]. Environmental compliance enables firms to attain operational efficiency in terms of meeting quality requirements and product specifications which improve the firm’s competitive advantage in the entire industry [46]. Recently, the supply chain has been greatly transformed where environmental compliance is the first consideration that suppliers check if they are to consider you as their supply chain partner or customer. This entails that firms which do not comply are likely to have outdated business models which augments chances of business failure. A study by De Jong, Paulraj and Blome [57] found that ISO 14001 certification improves the financial performance of a firm in both the short and long run. More importantly, the increase in financial performance was well pronounced for firms with ISO 14001 certification than those without.

On the other hand, other scholars report that adoption of international environmental standards such as ISO 4001 negatively affects financial performance [58]. Environmental compliance is costly to a firm as investments in technology and environmental compliance certificates drain the cash flows of a firm in the short run [59]. Boakye [46] investigated the relationship between environmental sustainability of firms listed on the Alternative Investment Market (AIM) in UK. The study used Small and Medium Enterprises as the sample. The results of the study revealed a negative relationship between environmental compliance and financial performance as measured by Tobin’s Q. Boakye [46] underscored that the negative relationship established between environmental compliance and financial performance might be because SMEs do not fully invest in environmental systems such as ISO 4001 and other environmental certifications. This inversely impacts on their efficiency which translates into a negative market valuation by potential investors. 

Opposing the above submissions, another line of thought argues that attaining environmental certification especially in the context of developing countries does not really add value to the firm [1]. The argument is that most investors are still driven by the profit maximization goal that the issue of environmental compliance is treated as secondary. On the other end of the spectrum, stakeholders such as customers react negatively to environmental certifications as they still perceive it as one of the marketing gimmicks used by firms to increase sales without necessarily adding value towards environmental protection [1]. Riaz et al. [1] further note that environmental policies in emerging markets are still weak and rarely enforced which makes most firms not to commit as they see their non-compliant counterparts doing well and unpunished. Boiral and Henri [60] noted that most firms adopt ISO 14001 certification as a strategy to gain legitimacy from the society. In fact, these firms do not implement the efficiency driven strategies as stipulated by the ISO 4001. The symbolic approach to ISO 4001 adoption can negatively affect the image of the firm as it is usually treated as deception and malpractice by stakeholders [61].

Even though there is divergence on the findings related to the relationship between environmental compliance and financial performance, the greater part of the empirical literature supports the view that there is a significant and positive relationship between environmental compliance and financial performance. The authors of this study also believe that firms can unlock financial benefits if they can comply with environmental policies. Essentially, firms can enhance their profitability by investing beyond just compliance as this can allow them to benefit from environmental investments such as ISO 14001 and internal environmental policies. Consistent with the Porter hypothesis, the authors of this study argue that environmental compliance can help listed firms to be proactive and find creative ways to reduce costs and boost their profitability as measured by EPS. The major argument derived from the Porter hypothesis is that environmental pollution is clear evidence that the firm’s operations and processes are highly inefficient. Additionally, if the firm incurs costs because of committing to environmental sustainability initiatives means the firm is doing it for compliance only for which it can achieve better cost savings benefits by being proactive and innovative. Firms which are viewed as legitimate can boost profitability by selling their green products at a premium. Furthermore, firms which excel in environmental compliance are likely to attract favorable ratings from the market which can boost the value of their shares and hence, an appreciation in their share price.

Hence, the following hypotheses will be tested in this study:

**Hypothesis** **Ha_3_.** *There is a significant positive relationship between environmental compliance and the EPS of firms listed on the JSE.*

**Hypothesis** **Ha_4_.** *There is a significant positive relationship between environmental compliance and the share price of firms listed on the JSE.*

## 2. Materials and Methods 

This study aimed to investigate the relationship between environmental sustainability commitment and financial performance. To attain this aim, the following research questions were formulated.

- Is there a relationship between carbon emission reduction and the EPS of firms listed on the JSE?
- Does carbon emission reduction significantly affect the share price of firms listed on the JSE?
- What is the nature of the relationship between environmental compliance and the EPS of firms listed on the JSE?
- Does environmental compliance significantly affect the share price of firms listed on the JSE?

The study area of this study was all FTSE/JSE listed firms. The researcher opted for a quantitative research approach and used a case study research design. This is because the researcher intended to collect data from several listed firms. A case study is defined as the assessment of a problem from a targeted set of organizations that are perceived to possess the characteristics of the problems being investigated [62]. A case study design was used following other similar studies such as Boakye [46] for consistency. The longitudinal design was adopted where the researcher collected panel data from 2011-2018. The reason for considering this period was that integrated financial reporting was introduced in 2010 in South Africa. Hence, relevant data was obtainable from 2011 and beyond [63]. All firms listed on the JSE were considered to be the population of this study. The choice to consider JSE listed firms was that these are critically scrutinized in terms of sustainability engagement and reporting [64].

### 2.1. Sample Selection

The sample of this study was 30 firms listed on the FTSE/JSE Responsible Investment Index. Nevertheless, 2 companies on the list had dual listings which made the number of firms to be 32 as shown in Table 1 below. The dual listing means the 2 public limited company (PLCs) are listed on the London Stock Exchange with a secondary listing in South Africa (JSE). These are company 11 PLC and 12 Ltd in the financial services sector as well as company 19 PLC and 20 Ltd in the manufacturing industry. This resulted in 256 observations for the period under consideration. The FTSE/JSE Responsible Investment Index is an index formed by the partnership between the JSE and the FTSE Russell in June 2015 in a bid to promote sustainable behavior among listed firms. Such a remarkable initiative was considered due to the rising demand for responsible investing. Hence, the collaboration between the JSE and the FTSE Russell aimed at enhancing environmental, social and governance (ESG) issues among listed firms and providing investors with in-depth information for them to make informed investment decisions. The FTSE/JSE consists of two indices which are the J113 and J110. The J113 consists of all firms which meet the listing requirements of the FTSE/JSE Responsible Investment Index which is 2.0 or above and whose market caps are evaluated daily. On the other hand, the J110 consists of the top 30 firms which excel in terms of ESG [64]. Hence, this study considered the top 30 FTSE/JSE firms as a suitable sample of this study. The firms on the FTSE/JSE Responsible Investment Index have been actively involved in sustainability practices. Additionally, they have satisfied the reporting requirements for both the FTSE and the JSE. This list was considered useful in the study because it is updated twice a year that is in June and December. This assisted the researcher to access all the data required to test the hypotheses of the study. This addressed the issue of missing data which usually causes problems in research [20]. To be included in the sample, the firm was supposed to meet the following criteria;

- The firm is currently listed on the FTSE/JSE Responsible Investment Index.
- The firm should have been actively reporting on environmental sustainability for the past 8 years.
- The firm’s integrated sustainability reports have data required for the study.

On the other hand, all newly listed JSE firms were excluded from the sample. These were removed because they could not meet the timeframe criteria and that the data required could be insufficient which could negatively impact the final results. The final list of the firms which were considered is showed below. To protect the image of the companies considered, the researchers ensured that there was no re-identification of firms considered by removing any form of identification such as the full or shortened name of the company. Instead, the researchers used firm codes ranging from 0-31 as shown in Table 1. 

Table 1 presents a list of top 30 firms listed on the FTSE/JSE Responsible Investment Index as of 18 June 2019. The list consists of 30 firms in their alphabetical order with an additional 2 firms due to dual listing. These are top performing firms in terms of ESG ratings. This list is reviewed regularly. This means that some firms exit while others join the list based on their ability to meet the ESG ratings. The firms on this list are from different industries as indicated in the table. Listed firms were considered because they contribute extensively towards environmental pollution from their business activities such as mining, construction, transport, and heating of offices. In terms of industry/sector category, 8 companies were from the mining industry, 4 in manufacturing, 3 in banking, 4 in health and pharmaceuticals, 4 in retail, 3 in telecommunications, 1 in energy, and 5 in services. There was no company from the agricultural and tourism sectors in the sample considered to be shown in Table 1. All the environmental policies of these listed firms are shaped by the Environmental Conservation Act 73 of 1989 which strongly encourage environmental protection while discouraging environmental pollution. This together with the listing requirements of the FTSE/JSE Responsible Investment influences environmental policies of the listed companies. The list presented above provides a rich source to collect data from as the researcher is guaranteed of quality findings and a fair representation of the status of environmental sustainability commitment among listed firms as more than one sector was considered.

### 2.2. Data Collection

This study used secondary data, which is annual financial statements of firms listed on the JSE. The data provided by these firms is critically audited to enhance transparency [17]. Secondary data is widely used in studies linking environmental sustainability to financial performance [17]. Financial performance data such as EPS and share price were collected from integrated annual financial statements on the firm’s websites and the McGregor database. Environmental sustainability commitment data related to carbon emission reduction and environmental compliance was collected from the listed companies’ sustainability reports and websites. Quantitative content analysis was used to collect data related to environmental sustainability variables such as carbon emission reduction and environmental compliance. Quantitative content analysis was used because it was difficult to find data related to the quantities of each unit of environmental sustainability variables such as carbon emission reduction and environmental compliance on the integrated annual reports. Unlike standardized financial performance measures, environmental sustainability variables such as carbon emission reduction and environmental compliance are still subjectively reported in most integrated annual reports of firms in South Africa. There is no uniformity in the reporting of these variables some firms report using the quantities of each while others report these variables subjectively. Hence, using content analysis to convert the subjective data to numerical data, the researchers developed key search words per each variable which were used to trace whether the variable was reported or not so that a score can be assigned to each outcome. The researchers used a dichotomous scale ranging from 0 and 1 for objectivity [65]. During data collection, a score of 0 was allocated when the variable was not reported and a score of 1 was allocated when the variable was reported in the financial year following similar studies [46,66]. 

Table 2 below was used to demonstrate the content analysis procedure followed by the researchers to collect environmental sustainability data. To that effect, 2 companies (a mining company with firm code 0 and a company with firm code 100 which operate in the health and pharmaceuticals) were used to demonstrate the content analysis procedure. It should be noted that this procedure was conducted on all the 32 firms in this study. To collect data related to carbon emission reduction, the researcher assessed reductions in direct emissions (scope 1), reductions in indirect emissions (scope 2) and investments in technology to trap CO_2_ and convert it into other economical uses. In this case, 0 was scored for a company which recorded an increase in carbon emissions by observing the reported metric tons of CO_2_ equivalent (mtCO2e) or subjective words such as “The increase in our carbon footprint was largely due to the inclusion of data from an additional 11 operations and from new services” which described an increase in carbon emissions in that year.” On the other hand, a score of 1 was allocated when the company recorded a decrease in carbon emissions compared to the previous year. The scores were then added together to get a total per each variable per year as indicated in Table 2. To collect data related to environmental compliance, a score of 0 was allocated when variables such as presence of ISO 14001, absence of fines and penalties, and presence of internal environmental policies were not attained while a score of 1 was allocated when the firm had all the variables mentioned above in place. All the subjective data was coded on Microsoft excel to quantify it and prepare it for further statistical tests in the Stata software.

Table 2 shows the procedure taken by the researchers during data collection using content analysis.

### 2.3. Data Analysis

Data was analyzed using the panel regression analysis model. The Feasible Generalized Least Squares model was adopted because the data had heteroskedasticity and serial auto correlation which could possibly cause problems in estimating the regression models [67]. Hence, the Feasible Generalized Least Squares model was used because it suppresses both heteroskedasticity and serial autocorrelation [67].

### 2.4. Measurement of Variables 

#### 2.4.1. Dependent Variable (Financial Performance)

The dependent variable of the study was financial performance. This study adopted both the accounting and market-based measures of financial performance. The use of both measures can strengthen the predictive and explanatory power of a model [68] which improves the accurateness of the financial performance construct [69,70]. This has been lacking among studies conducted in South Africa regarding environmental sustainability commitment and financial performance nexus.

##### EPS

According to Madugba and Okafor [71], EPS shows the amount of earnings allocated to shareholders. EPS was considered an important ratio in this study because firms listed on the JSE are required to publish their EPS. More importantly, EPS is used for strategic decisions such as stock valuations and possibilities of considering another firm for possible joint ventures or mergers. Ideally, EPS is also relatively easy to calculate, hence, making it easier for investors to understand it [72]. 

EPS is calculated as follows;

(1) $$\mathrm{EPS}\;=\frac{\mathrm{Profit}\;\mathrm{or}\;\mathrm{loss}\;\mathrm{attributable}\;\mathrm{to}\;\mathrm{ordinary}\;\mathrm{shareholders}\;\mathrm{of}\;\mathrm{the}\;\mathrm{parent}\;\mathrm{firm}}{\mathrm{Weighted}\;\mathrm{average}\;\mathrm{number}\;\mathrm{of}\;\mathrm{ordinary}\;\mathrm{shares}\;\mathrm{issued}},$$

##### Share Price

A share price measures the value of the business [73]. It is a crucial firm performance metric assessed by investors before they can invest in a business. A higher share price implies that the concerned firm is highly valued by the market [74]. 

#### 2.4.2. Independent Variable/s (Environmental Sustainability Commitment)

The independent variables of the study were components of environmental sustainability. These were carbon emission reduction and environmental compliance. The environmental sustainability variables used in this study were derived from the Global Reporting Initiative (GRI) guidelines (EN category). The GRI indicators have been used widely as guidelines on measures of sustainability [17,46]. Each of the environmental sustainability variable is presented below. 

##### Carbon Emission Reduction

In this study, carbon emission reduction was measured by assessing reductions in direct emissions (scope 1), reductions in indirect emissions (scope 2) and investments in technology to trap CO_2_ and convert it into other economical uses. Carbon emission is measured using metric tons of CO_2_ equivalent (mtCO2e). The data was sourced from sustainability reports of each firm considered. The sustainability reports are published on each firm’s website annually as mandated by the JSE. This was done using the strict guidelines provided by the Global Reporting Initiative. This was done following other similar studies such as Boakye [46] for consistency. 

##### Environmental Compliance

Environmental compliance was measured based on factors such as obtaining environmental certification, investment in international standards such as ISO 14001, absence of fines and penalties, and presence of internal environmental policies. The data was sourced from sustainability reports of each firm considered. This was done using the strict guidelines provided by the Global Reporting Initiative. This was done following other similar studies such as Boakye [46] for consistency.

Dependent variable; Y: Financial performance
  Dependent variable 1; Y: Earnings per share (EPS)
  Dependent variable 2; Y: Share price
Independent variable; X: Environmental sustainability
  Independent variable 1; X1: carbon emission reduction
  Independent variable 2; X2: environmental compliance
Panel regression model
  Y_it_ = α + X_1it_ + X_2it_+X_3it_ + X_4it_ + ε (2)
  where y = financial performance; x_1_ = carbon emission reduction (cer); x_2_ = environmental compliance; x_3_ = firm size; x_4_ = Liquidity; +ε = error term; α = constant.
Control variables

It is important to determine if other underlying factors have an influence on the dependent variable [75]. Hence, these factors should be tested prior to the independent variable to provide an alternate explanation for the findings. Control variables such as firm size and liquidity were used following similar studies [68,76]. This is because the size of the firm and liquidity influence the overall financial performance of a firm [77,78]. In this study, market capitalization was used to measure the size of the firm while liquidity was measured by compiling values from the current ratio of firms which were evaluated. 

## 3. Results

### 3.1. Descriptive Statistics 

Table 3 presents descriptive statistics for key variables of the study. As indicated in Table 3, the total number of observations was 256 derived from 32 firms observed for 8 years. Carbon emission reduction had a mean of 2.253906 and a standard deviation of 0.942454. In terms of environmental compliance, the mean score was 2.785156 and the standard deviation was 0.411518. As indicated by Table 1, the minimum value for all environmental variables was 0 and the maximum was 1. In terms of EPS, the mean score was 1181.074 and the standard deviation was 1385.127, while the minimum value was −1764.32 and the maximum was 12,044.82. It is also noted that share price ranged from a minimum value of 0 to a maximum of 86,734. The findings show that the mean for liquidity was 1.425118 and the standard deviation was 0.9830142. The minimum value for liquidity was 0 and the maximum value was 6.8176. Considering firm size, the mean score was 929,723 and the standard deviation was 47,711.28. The minimum value was 0 and the maximum value was 428,668. 

### 3.2. Correlation Analysis

Table 4 shows correlation analysis results among variables. The results showed that carbon emission reduction was positively correlated with EPS (0.149) and share price (0.1442). Also, a positive relationship was established between environmental compliance and EPS (0.1955) as well as with share price (0.2121). The findings also showed that carbon emission reduction was positively correlated with liquidity (0.0777) and with firm size (0.0688) even though the correlation was weak. Moreover, environmental compliance was found to be positively correlated with liquidity (0.0465) and negatively correlated with firm size (−0.1388).

### 3.3. Environmental Sustainability Commitment and the EPS Model

Table 5 presents findings on the relationship between environmental sustainability variables and financial performance based on the EPS model. A positive and significant relationship (284.074; sig 0.001) was established between carbon emission reduction and EPS. Also, a positive and significant relationship (545.76; sig 0.007) was established between environmental compliance and EPS. This suggests that being a compliant business in terms of environmental requirements such as ISO 14001 and internal environmental policies may increase the profitability of the business as measured by EPS. Based on the results presented in Table 5, the alternative hypothesis stating that **t**here is a significant positive relationship between carbon emission reduction and the EPS of firms listed on the JSE was supported and accepted leading to the rejection of the null hypothesis. Additionally, the alternative hypothesis stating that **t**here is a significant positive relationship between environmental compliance and EPS of firms listed on the JSE was supported and accepted leading to the rejection of the null hypothesis. The implication of the above results is that environmental sustainability commitment is a crucial determinant of financial performance of firms listed on the JSE as measured by EPS especially among the industries considered in this study such as the mining industry, manufacturing, banking, health and pharmaceuticals, retail, telecommunications, energy, and the services sector.

### 3.4. Environmental Sustainability Commitment and the Share Price Model

Table 6 shows findings on the relationship between environmental sustainability variables and financial performance. A significant positive relationship (2807.732; sig 0.003) was established between carbon emission reduction and share price. Additionally, a positive and significant relationship (5493.066; sig 0.014) was established between environmental compliance and share price. Based on the results presented in Table 6, the alternative hypothesis stating that **t**here is a significant positive relationship between carbon emission reduction and share price of firms listed on the JSE was supported and accepted leading to the rejection of the null hypothesis. Additionally, the alternative hypothesis stating that there is a significant positive relationship between environmental compliance and share price of firms listed on the JSE was supported and accepted leading to the rejection of the null hypothesis. Based on the findings presented above, one can infer that environmental sustainability commitment variables such as carbon emission reduction and environmental compliance enhances the financial performance of listed firms when the share price is considered. The implication of this is that listed firms can actually enhance their market value by investing and committing towards environmental sustainability initiatives as confirmed by the findings of this study. 

## 4. Discussion

### 4.1. Carbon Emission Reduction and Financial Performance

The findings showed that there is a significant positive relationship between carbon emission reduction and financial performance. This suggests that with the emergence of green investors, it has become imminent for firms to consider carbon emission reduction seriously. The results provide enough evidence that carbon emission reduction does not only affect EPS, but it also positively influences the share price. Hence, firms which actively invest in initiatives aimed at reducing carbon emissions are likely to experience superior financial performance. This is because it is now crucial than ever to excel in environmental performance. For instance, in South Africa it is now mandatory for firms listed on the JSE to disclose their environmental impact and different measures employed to mitigate such. To show the seriousness of environmental responsible behavior, the FTSE/JSE responsible investment index have been established to continuously evaluate firms on their ESG performance. The findings of this study agree with existing similar studies. For instance, a study by Fujii et al. [79] established that improved environmental performance leads to an improved financial performance. Similarly, Charlo, Moya and Muñoz [80] also discovered that firms with a positive environmental performance report improved financial performance. Using large Italian firms, Cucchiella et al. [39] found that cutting down carbon emissions has beneficial effects on the financial performance of a firm. This was supported by Yu and Tsai [47] who alluded that efforts by a firm to cut down its carbon emissions improve the longevity and sustainability of the firm. Lewandowski [48] also found a significant positive relationship between carbon emission reduction and financial performance. The results further indicated that the relationship between carbon emission reduction and financial performance was stronger for companies which excelled in carbon emission reduction initiatives compared to the laggards. Capece et al. [49] also indicated that firms actively involved in initiatives to cut down emissions showed positive financial performance. Low levels of carbon emissions are associated with higher firm financial performance [81]. Essentially, low levels of carbon emissions might also mean that the firm’s operations are efficient which cut costs in all areas within the firm leading to superior financial performance [82]. The cost savings can be because the firm can now operate without environmental penalties and lawsuits by environmental pressure groups. 

### 4.2. Environmental Compliance and Financial Performance

A positive and significant relationship between environmental compliance and all financial performance measures was established. This suggests that being a compliant business in terms of environmental requirements such as ISO 14001 and internal environmental policies may increase the profitability of the business as measured by EPS. Moreover, the implication of the positive and significant relationship between environmental compliance and share price is that investors value companies which comply with environmental regulations. This gives them assurance that the business will continue to have a positive image which protects their stake in the firm. Share price is highly sensitive to bad publicity of the business which may emerge because of lack of compliance. Hence, a firm is likely to avoid penalties and fines if they comply with environmental policies and standards such as ISO 4001 standards. Environmental compliance enables firms to attain operational efficiency in terms of meeting quality requirements and product specifications, which improve the firm’s competitive advantage. The findings above are supported by a plethora of other similar existing studies. For instance, Porter [83] argues that strong and stringent environmental policies force firms to fully commit towards environmental protection. To that effect, a well-structured environmental legislation makes it possible for firms to be profitable while at the same time being environmentally responsible. The Porter hypothesis takes a “revisionist” approach which opposes the widely held views by the traditionalist approach which argues that environmental legislation can weaken the financial performance of a firm. To that effect, Porter [83] is of the view that environmental legislation can force firms to innovate which translates into environmental sustainability commitment. In that case, a firm can benefit from new product innovations and systems innovation which helps the firm to be a market leader, hence, motivates it to invest in environmental sustainability initiatives. New innovations can also help a firm meet and surpass its financial goals by cutting costs. A firm is likely to avoid costs which come with fines and penalties emanating from non-compliance [46,56]. Even though the cost of maintaining an environmentally compliant status might be relatively high, fines and penalties emanating from environmentally irresponsible behavior can be unbearable [54]. For instance, BP is still haunted by a case emanating from the 2010 oil spill in the Gulf [55]. Such a penalty can leave a firm with serious cash flow problems. A study by De Jong et al. [57] also found that environmental compliance improves the financial performance of a firm in both the short and long run. Essentially, environmental compliance enhances the legitimacy of the business in the perspective of the community which can boost its corporate image [84]. Ideally, firms with a positive image are likely to witness an increase in the value of their shares, hence, a favourable financial performance. 

## 5. Conclusions

Essentially, this study aimed at examining the relationship between environmental sustainability commitment and financial performance of firms listed on the JSE. Broadly, the researcher aimed to establish whether environmental sustainability activities such as carbon emission reduction and environmental compliance do affect financial performance as measured by EPS and share price. The objectives were empirically tested using the panel regression model. Specifically, the Feasible Generalized Least Squares was adopted. It was concluded that firms can enhance their financial performance from environmental investment as supported by a significant and positive relationship established between environmental sustainability variables and both accounting (EPS) and market-based (share price) measures of financial performance. The study makes several recommendations based on the existing state of environmental sustainability commitment among listed firms. For instance, strict environmental legislation at national level is required to force firms to take environmental sustainability commitment seriously. A perfectly structured legal environment can ignite innovation among firms which can enable them to attain environmental sustainability yet profitable. It is pertinent to note that firms need to account for their environmental damage by changing their attitude towards the environment which can enable them to adopt proactive environmental sustainability strategies. For firms to optimize their gains from environmental protection, the authors of this study suggest that they should invest beyond just compliance and find different innovative combinations which can reward their investments positively. Another crucial point is that environmental sustainability commitment should not be treated as a once off event but should be viewed as a continuous process which requires listed firms to become learning organizations in terms of environmental sustainability commitment. This study contributed new empirical evidence that environmental sustainability initiatives such as carbon emission reduction and environmental compliance significantly predict financial performance especially among firms operating in the mining industry, manufacturing, banking, health and pharmaceuticals, retail, telecommunications, energy, and the services sector. The practical implication of the study is that the findings of this study can help managers of firms listed on the JSE in South Africa to adopt proactive ways to reduce carbon emissions and craft internal environmental policies as the study established that such a strategy positively influences financial performance. Essentially, the findings of this study could be useful in shaping the environmental policy framework in South Africa to mitigate the environmental sustainability conundrum. Even though the study was successful in achieving its objectives, it had a few limitations. One key limitation is that several factors not just environmental sustainability commitment can affect EPS and share price of the listed firms. Thus, the study only assessed the effect of carbon emission reduction and environmental compliance on EPS and share price. The limitations of this study can be improved by future studies which may explore other factors that can affect EPS and share price. Other future studies can assess the effect of slack resources such as financial resources on environmental sustainability commitment among listed firms on the JSE with a focus on other industries such as agriculture, tourism, and construction not covered in the current study.

## Figures and Tables

**Table 1:** List of firms listed on the FTSE/JSE Responsible Investment Index.

Statistic Date	Firm Code	Industry
2018/06/18	0	Mining
2018/06/18	1	Mining
2018/06/18	2	Mining
2018/06/18	3	Mining
2018/06/18	4	Banking
2018/06/18	5	Mining
2018/06/18	6	Manufacturing
2018/06/18	7	Health and pharmaceuticals
2018/06/18	8	Retail
2018/06/18	9	Mining
2018/06/18	10	Mining
2018/06/18	11	Financial services
2018/06/18	12	Financial services
2018/06/18	13	Services
2018/06/18	14	Mining
2018/06/18	15	Health and Pharmaceutical
2018/06/18	16	Retail
2018/06/18	17	Health and Pharmaceutical
2018/06/18	18	Insurance and Financial services
2018/06/18	19	Manufacturing
2018/06/18	20	Manufacturing
2018/06/18	21	Telecommunications
2018/06/18	22	Banking
2018/06/18	23	Health and Pharmaceutical
2018/06/18	24	Financial services
2018/06/18	25	Manufacturing
2018/06/18	26	Oil and gas Chemical
2018/06/18	27	Banking
2018/06/18	28	Telecommunications
2018/06/18	29	Retail
2018/06/18	30	Telecommunications
2018/06/18	31	Retail

Source: Johannesburg Stock Exchange (JSE) [64].

**Table 2:** Content Analysis Procedure.

**Firm Code 0**	**2011**	**2012**	**2013**	**2014**	**2015**	**2016**	**2017**	**2018**
Carbon emission reduction								
Reductions in direct emissions (scope 1)	1	1	1	1	1	1	1	1
Reductions in indirect emissions (scope 2)	1	1	1	1	1	1	1	1
Investments in technology to trap CO_2_	1	1	1	1	1	1	1	1
Total	3	3	3	3	3	3	3	3
Environmental compliance								
Presence of ISO 14001	1	1	1	1	1	1	1	1
Absence of fines and penalties	1	1	1	1	1	1	1	1
Internal environmental policies	1	1	1	1	1	1	1	1
Total	3	3	3	3	3	3	3	3
**Firm Code 17**	**2011**	**2012**	**2013**	**2014**	**2015**	**2016**	**2017**	**2018**
Carbon emission reduction								
Reductions in direct emissions (scope 1)	0	1	0	0	1	1	0	1
Reductions in indirect emissions (scope 2)	0	1	0	0	1	0	0	1
Investments in technology to trap CO_2_	1	1	1	1	1	1	1	1
Total	1	3	1	1	3	2	1	3
Environmental compliance								
Presence of ISO 14001	1	1	1	1	1	1	1	1
Absence of fines and penalties	1	1	1	1	1	1	1	1
Internal environmental policies	1	1	1	1	1	1	1	1
Total	3	3	3	3	3	3	3	3

Source: The authors’ own compilation.

**Table 3:** Descriptive statistics.

Variable	Obs	Mean	Std. Dev.	Min	Max
Carbon Emission Reduction	256	2.253906	0.942454	0	3
Environmental Compliance	256	2.785156	0.411518	0	3
EPS	256	1181.074	1385.127	−1764.32	12,044.82
Share Price	256	15,695.86	14,525.73	0	86,734
Liquidity	256	1.425118	0.9830142	0	6.8176
Firm Size	256	929,723	47,711.28	0	428,668

**Table 4:** Correlation Analysis.

Variables	EPS	Share Price	Emissions	Compliance
EPS	1			
Share Price	0.7843	1		
Carbon emission reduction	0.149	0.1442	1	
Compliance	0.1955	0.2121	0.1008	1
Liquidity	0.0241	0.1333	0.0777	0.0465
Firm size	0.1721	−00061	0.0688	−0.1388

**Table 5:** Model 1 Feasible Generalized Least Squares (FGLS) regression—EPS.

Cross sectional Time Series FGLS Regression							
Coefficients: Generalized Least Squares							
Panels:	Heteroskedastic						
Correlation: Common AR (1) Coefficient for All Panels (0.0298)							
Estimated covariances		=	8		Number of obs=		256
Estimated autocorrelation		=	1		Number of groups=		8
Estimated coefficients		=	11		Time periods=		32
					Wald chi2 (10)=		63.67
					Prob >chi2=		0.0000
EPS	Coef.	Std. Err.	z	*p* > |z|	[95% confi.		Interval]
Emissions	284.074	85.54287	3.32	0.001	116.4131		451.735
Compliance	545.7638	204.024	2.67	0.007	145.8841		945.6435
Liquidity	238.6154	73.12317	3.26	0.01	95.29661		381.9342
Firm size	0.0015856	0.001781	0.89	0.373	−0.0019058		0.005077
_cons	−0.2044149	1183.034	−1.73	0.084	−4362.852		274.5548

**Table 6:** Model 2 FGSL regression—share price.

Cross sectional Time series FGLS Regression							
Coefficients: Generalized Least Squares							
Panels:	Heteroskedastic						
Correlation: Common AR (1) Coefficient for All Panels (0.0298)							
Estimated covariances		=	8		Number of obs=		256
Estimated autocorrelation		=	1		Number of groups=		8
Estimated coefficients		=	11		Time periods=		32
					Wald chi2 (10)=		75.18
					Prob > chi2=		0.0000
Share Price	Coef.	Std. Err.	z	*P* > |z|	[95% confi.		Interval]
Emissions	2807.732	939.4598	2.99	0.003	966.4246		4649.039
Compliance	5493.066	2238.029	2.45	0.014	1106.61		9879.523
Liquidity	1281.386	793.505	1.61	0.106	−273.8551		2836.628
Firm size	0.0189978	0.087419	1.01	0.311	−0.0177356		0.0557313
_cons	12,391.2	12,990.94	0.95	0.340	−13070.58		3785299
